# The Relationship Between Indicators of Nasal Respiratory Function and Spirometric Parameters in Children With Bronchial Asthma

**DOI:** 10.3389/fped.2020.580043

**Published:** 2021-01-14

**Authors:** Svetlana V. Krasilnikova, Alexey A. Khramov, Regina N. Khramova, Dmitry Yu. Ovsyannikov, Mojisola I. Daniel-Abu, Alexey Novozhilov, Andrey V. Shahov, Nailya I. Kubysheva, Tatyana I. Eliseeva

**Affiliations:** ^1^Department of ENT Diseases, Federal State Budgetary Educational Institution of Higher Education “Privolzhsky Research Medical University” of the Ministry of Health of the Russian Federation, Nizhny Novgorod, Russia; ^2^Department of Hospital Pediatrics, Federal State Budgetary Educational Institution of Higher Education “Privolzhsky Research Medical University” of the Ministry of Health of the Russian Federation, Nizhny Novgorod, Russia; ^3^Department of Pediatrics, Peoples' Friendship University of Russia, Moscow, Russia; ^4^ENT-Department, Volga District Medical Center Federal Medical-Biological Agency Russia, Nizhny Novgorod, Russia; ^5^Clinical Linguistics Laboratory, Kazan Federal University, Kazan, Russia

**Keywords:** bronchial asthma, allergic rhinitis, rhinomanometry, spirometry, children

## Abstract

**Introduction:** The relationship between objective indicators of nasal obstruction and airflow limitation in children with bronchial asthma (BA) and allergic rhinitis (AR) has not yet been studied.

**Objective:** To study the relationship between objective parameters of nasal obstruction and airflow limitation, determined using the methods of anterior active rhinomanometry (AARM) and spirometry in children with BA and AR.

**Materials and Methods:** Eighty eight children and adolescents with BA and AR, boys−65.9% (58/88), were examined. The median age was 11.09 [10.42; 11.76] years. To determine airflow limitation, the following spirometric parameters were evaluated: forced vital capacity of the lungs (FVC), forced expiratory volume in 1 s (FEV_1_), the ratio of FEV_1_/FVC, and maximum expiratory flow at the point 25% of the flow-volume loop (MEF_25_). Data were recorded both in absolute values and in relative units (% pred). Nasal respiratory function was determined by AARM based on the total nasal airflow (TNAF) in absolute (Pa/cm^3^/s) and relative units (RTNAF, % pred).

**Results:** In the general cohort and in boys but not in girls, a statistically significant direct correlation was found between TNAF (Pa/cm^3^/s) and absolute spirometry parameters of bronchial patency—all had *p* < 0.01. Also, RTNAF and relative MEF_25_ values (% pred) in the general cohort were *R* = 0.22, *p* = 0.04, and in boys, *R* = 0.28, *p* = 0.03. In girls, there was no statistically significant correlation between nasal respiratory function and spirometric parameters, all *p* > 0.05. Additional analysis of literature was conducted to ascertain that the identified gender differences were not occasional.

**Conclusion:** The significant positive correlation of absolute values of AARM and spirometric parameters in children with BA and AR was established, which apparently reflects the physical development of children. Of all the relative indicators of spirometry, only MEF_25_ (% pred), which indirectly reflects the patency of small bronchi, had a distinct direct correlation with RTNAF. These patterns are clearly expressed in boys with BA. In girls with this disease, however, the relationship between nasal respiratory function and spirometric indicators seems to be more complex and requires further study.

## Introduction

Bronchial asthma (BA) is the most common chronic respiratory disease in children characterized by bronchial hypersensitivity in combination with variable bronchial obstruction. Currently, the goal of the treatment of this disease is to achieve control over the symptoms and risk factors for exacerbation of the disease (1, 2). This is realized by the basic anti-inflammatory therapy since the most significant component of the pathogenesis is chronic inflammation localized in the respiratory tract and occurring in childhood mainly by the Th2-dependent mechanism (3–7). This approach to the BA treatment has shown significant success. However, modern research demonstrates that, despite an arsenal of pharmacological agents, the proportion of patients who do not achieve proper control may be up to 56% (4, 8).

One of the reasons for the insufficient level of BA control is the negative impact of comorbidities on the course of BA, primarily upper respiratory tract (URT) pathologies, including allergic rhinitis (AR) and allergic rhinosinusitis (ARS) (9–14). IgE-mediated inflammation of the nasal mucosa within the structures of the ostiomeatal complex leads to impairment of mucociliary transport, drainage of mucus from the paranasal sinuses, as well as to ventilation disorders (15, 16). This, in turn, may have a negative effect on the lower respiratory tract due to the development of rhinobronchial reflex and cytokinemia (17). The commonness of the development of the inflammatory process in the upper and lower respiratory tract mucosa in BA is reflected in the concept of “one airway—one disease” (18).

The prevalence of AR in patients with asthma is high (16). In our study, it was previously demonstrated that AR may occur in all children with atopic BA who have nasal symptoms (19), which is consistent with the results reported in Blaiss (20). The use of modern imaging methods enabled to identify anomalies of intranasal structures, hypertrophic changes in the pharyngeal tonsil, and debuting hypertrophy of the nasal mucosa in a significant fraction of children with atopic BA (12, 13, 19). This indicates that the processes of remodeling of the extracellular matrix are involved in the formation of pathological changes in the respiratory tract in these patients (21).

In recent years, there have been publications indicating that, despite similar mechanisms of allergic inflammation, the URT and LRT have different mechanisms of pathological remodeling responding differently to damaging stimuli (22–24). This is probably due to their different embryonic origins (the structures of the nasal cavity originate from the ectoderm, whereas LRT from the endoderm) which result in the different morphological and functional manifestations of pathological remodeling. This corrects the “One airway—one disease” concept (25).

Despite numerous publications showing the association of upper and lower airway pathology in patients with BA, studies on the direct measurement and comparison of upper and lower airflow limitation in patients with BA are few (26). This makes it difficult to understand the mechanism of the relationship between the pathology of the URT and LRT in patients with BA.

Spirometry is a reliable method for assessing expiratory airflow limitation, and has gained a strong practical position in the management of patients with asthma (27). The main spirometric indicators for assessing bronchial obstruction are forced expiratory volume in 1 second (FEV_1_) and the Tiffeneau index, defined as the ratio of FEV_1_ to forced vital capacity (FVC) (27). Spirometric data of patients are evaluated in comparison with the range of their values for healthy people, taking into account age, height, gender and race (3, 27). In addition, airway patency is reflected by the flow-volume loop, namely, flow at points 75, 50, and 25% of the exhaled FVC. It is believed that the parameters of the flow-volume loop are more reproducible and more sensitive than FEV_1_ for detecting “small respiratory tract disease” (28, 29).

When determining nasal respiratory function, the “gold standard” is considered to be anterior active rhinomanometry (AARM), which is a highly sensitive method for studying nasal breathing. There is evidence that AARM indicators, as well as spirometry indicators, depend on the age, gender, and anthropometric data of patients. However, the range of normal values of AARM indicators in healthy people cannot yet be considered established, since these data have so far been obtained only on limited samples (30–32).

Discussion on the similarities and differences of pathological process in URT and LRT in patients with respiratory allergies can be resolved by comparing the air flow characteristics in different regions of the respiratory tract. Studies of this kind, however, are few. We found only three studies that included objectification of both nasal and bronchial patency in patients with BA and nasal symptoms. Ciprandi et al. (26) demonstrated the relationship between nasal and bronchial respiratory functions in adult patients with perennial allergic rhinitis and asthma. Motomura et al. (33) found in their study the combined limitation of airflow in the upper and lower airways in patients with AR. Iyer and Athavale (34) reported a high risk of latent damage to the small airway in patients with AR (30). Numerous other studies demonstrating the relationship between the severity of rhinitis symptoms and the course of asthma are usually not supported by combined functional studies of nasal and bronchial respiratory function (13, 35, 36). Thus, the relationship between objective indicators of nasal and bronchial respiratory functions in children with BA and nasal symptoms is still insufficiently studied.

Therefore, the aim of this research was to study the relationship between nasal and bronchial patency in children with BA and AR using the methods of AARM and spirometry.

## Materials and Methods

### Formation of Cohort of Patients

The study was carried out in accordance with the Helsinki Declaration, adopted in June 1964 (Helsinki, Finland) and revised in October 2000 (Edinburgh, Scotland). The study was approved by the Ethics Committee of the Privolzhsky Research Medical University, Protocol No. 13, dated 10.10.16. Informed consent was obtained from patients aged 15–17 years and from parents of patients aged less than 15 years, in accordance with Federal Law No. 323 dated 21.11.2011 “On the basics of public health protection in the Russian Federation.”

Eighty eight children and adolescents aged 6 to 17 years, who were treated for the atopic BA in the 1st Children's City Clinical Hospital of Nizhny Novgorod, between years 2018 and 2019, and had sinonasal complaints, such as nasal obstruction, nasal discharge, headache, sneezing and itching of the nose, were examined. Their median age was 11.09 [10.42; 11.76] years; boys made up 65.9% (58/88) of the patients. Verification of BA and AR was done in accordance with existing domestic and international conciliation documents (1).

All the children were found to have symptoms characteristic of BA and AR. The family history associated with atopy (asthma, AR, conjunctivitis, atopic dermatitis, urticaria) was evaluated, and positive skin test results were obtained, or high titers of specific class E immunoglobulins were detected for at least one of the most common aeroallergens in the Volga-Vyatski region of the Russian Federation (37).

Criteria for inclusion were a diagnosis of BA, made in accordance with current international and national conciliatory documents, and the presence of nasal or sinonasal complaints and symptoms in patients (1). Exclusion criteria were: acute infectious diseases and fever, diabetes, autoimmune disorders, primary immunodeficiency, and cancer, as well as oral glucocorticoid intake (38). The diagnosis of BA and the severity of the disease were established by the attending physician in accordance with the recommendations available at that time (GINA 2017-2019) (1). The treatment of BA was carried out in accordance with existing conciliation documents, taking into account modern therapeutic strategies (1, 4, 39).

### Objective and Subjective Measurements

All children had a general clinical examination. Due to the presence of nasal symptoms, all patients were examined by an experienced otorhinolaryngologist with a routine otolaryngological examination.

A quantitative assessment of the level of BA control was performed using the Asthma Control Questionnaire-5 (ACQ-5) test. Test values from 0 to 0.75 corresponded to achieved control of BA, from 0.75 to 1.5 to partial control, and 1.5 points or higher—the absence of disease control (40, 41). The severity of nasal symptoms was quantified using the total nasal score symptoms (TNSS) scale, which includes four questions (severity of rhinorrhea, sneezing, itching, and nasal obstruction). Each symptom is evaluated on a 4-point scale from 0 points (no symptom) to 4 points (the greatest severity of the symptom), followed by the determination of the overall score (42).

Assessment of nasal breathing function—Respiratory function of the nose was assessed by the AARM method using a computer rhinomanometer—Rhino 31 (Atmos, Germany) in accordance with the available recommendations (43). Computer software allowed to obtain parameters of the volume flow of inhaled air passing through the right and left halves of the nose, the total nasal airflow (TNAF). The TNAF was automatically calculated at a pressure of 150 Pa/cm^3^/s (31). In order to take into account the age and gender differences, we also used the “relative total nasal airflow” index (RTNAF, %pred) introduced earlier (14) according to the formula RTNAF = TNAF/ETNAF × 100%, where TNAF—indicators of the patient's TNAF (absolute units), ETNAF—expected values of TNAF for age, gender and anthropometrical indicators elaborated in Eliseeva et al. (14) and Zapletal and Chalupova (31).

Rhinomanometric studies were performed in the morning hours. Twenty four hours prior to this procedure, intranasal corticosteroids and decongestants were canceled. It was performed in a sitting position, one nostril of the patient was completely covered with a special nasal adaptor, and the patient was asked to breathe calmly and evenly through a silicone half-mask with a closed mouth. The measurement results were displayed in real-time as a rhinogram (“respiratory loops” according to Bachmann) (44), and after the measurement was completed, as a diagram stored in the computer memory.

Spirometrics studies were performed using MasterScreen pneumatic spirometer (Jaeger, Germany) in accordance with international recommendations (27, 45). The forced vital capacity (FVC), forced expiratory volume in 1 s (FEV_1_), ratio FEV_1_/FVC, the maximum expiratory flow rate at points 75% (MEF_75_) and 25% (MEF_25_) of the “flow-volume” loop, which reflect the flow velocity at the proximal and distal segments of the loop, respectively, were also evaluated. Data were recorded both in absolute values of indicators and in relative units (in comparison with the expected values for the age, gender, and anthropometric indicators of the child) (46). Additionally, tests for the reversibility of bronchial obstruction were also performed in patients, but their results were not included in this work, a separate study will be devoted to their analysis.

### Statistical Analysis

The study was a pilot, so the required sample size was not calculated. The statistical analysis was performed using the Statgraphics Centurion V. 16.1.17 software package. The median and interquartile range were calculated for each of the quantitative indicators. The data are presented as Me [Q1; Q3], where Me is the median, and [Q1; Q3] is the interquartile range.

Checking samples for normality—for quantitative features, standardized skewness and standardized kurtosis were calculated to determine the normality of the sample. If the calculated values of standardized skewness and standardized kurtosis went beyond the range from −2 to +2, then the quantitative samples in question were considered to be different from normal. Based on the calculations, a normal distribution was observed in the following samples: age; height; TNSS (balls); FVC (% pred); FEV_1_ (% pred); MEF_75_ (% pred); MEF_25_ (% pred); TNAF (Pa/cm^3^/s); RTNAF (% pred). Distribution different from normal was found in these samples: ACQ-5 (balls), FVC (l), FEV_1_ (l), FEV_1_/FVC ratio, MEF_75_ (l/s), MEF_25_ (l/s).

The differences between the two groups were determined using the Student's *t*-test criteria to compare the means of the two samples if the sample distribution was normal, and using the Wilcoxon Mann-Whitney W-test to compare the medians of the two samples (for sample distributions different from normal) and KWT-test in order to compare medians of several samples. The relationship between the indicators was evaluated using Spearman's rank correlation. The value *p* < 0.05 was taken as the level of statistical significance.

## Results

### Clinical Features of Patients

The analysis was done both in the general cohort and separately among boys and girls.

The average age of children was 11.09 [10.42; 11.76] years; boys and girls were comparable in age. The age and height of patients had no gender differences (Table 1).

**Table 1:** Clinical and functional characteristics of examined children with BA.

**Parameters**	**All**	**Boys**	**Girls**	**Statistics**
Number of patients	88	58	30	-
Age, years	11.09 [10.42; 11.76]	10.97 [10.21; 11.72]	11.33 [9.96; 12.71]	*p* = 0.61
Height, cm	151.20 [147.56; 154.83]	152.53 [148.08; 156.97]	148.63 [142.03; 155.23]	*p* = 0.32
BMI	19.32 [18.30; 20.33]	19.46 [18.29; 20.63]	19.04 [16.99; 21.08]	*p* = 0.34
ACQ-5, balls	0.72 [0.52; 0.91]	0.74 [0.49; 0.99]	0.67 [0.36; 0.98]	*p* = 0.86
TNSS	3.16 [2.63; 3.70]	3.27 [2.56; 3.97]	2.96 [2.13; 3.97]	*p* = 0.60
FVC, l	3.27 [3.05; 3.49]	3.41 [3.13; 3.69]	3.01 [2.65; 3.37]	*p* = 0.13
FVC, % pred	109.80 [106.81; 112.78]	108.49 [104.66; 112.32]	112.32 [107.47; 117.18]	*p* = 0.23
FEV_1_, l	2.48 [2.29; 2.67]	2.57 [2.32; 2.82]	2.31 [2.05; 2.57]	*p* = 0.34
FEV_1_, % pred	98.94 [95.27; 102.60]	97.09 [92.55; 101.63]	102.51 [96.13; 108.87]	*p* = 0.16
FEV_1_/FVC (%)	75.92 [73.69; 78.15]	74.69 [72.03; 77.34]	78.31 [74.17; 82.45]	*p* = 0.13
MEF_75_, l/s	4.01 [3.65; 4.37]	4.16 [3.66; 4.65]	3.72 [3.26; 4.18]	*p* = 0.52
MEF_75_, %pred	82.37 [77.30; 87.45]	83.00 [76.37; 89.63]	81.17 [73.07; 89.27]	*p* = 0.74
MEF_25_, l/s	1.11 [0.96; 1.26]	1.11 [0.91; 1.31]	1.10 [0.90; 1.31]	*p* = 0.54
MEF_25_, % pred	61.62 [55.49; 67.74]	58.91 [51.45; 66.37]	66.85 [55.79; 77.91]	*p* = 0.21
TNAF, Pa/cm^3^/s	610.41 [565.70; 655.11]	589.26 [534.23; 644.29]	651.30 [572.23; 730.37]	*p* = 0.19
RTNAF, % pred	94.51 [87.76; 101.26]	89.90 [82.33; 97.48]	103.42 [90.02; 116.81]	*p* = 0.06

*BMI, kg/m^2^–body mass index; ACQ-5, balls—the Asthma Control Questionnaire-5; TNSS, balls—the total nasal score symptoms scale; FVC, l—forced vital capacity of the lungs; FVC, % pred—relative forced vital capacity of the lungs; FEV_1_, l—forced expiratory volume in 1 s; FEV_1_, % pred—relative forced expiration volume in 1 s; MEF_75_, l/s—maximum expiratory velocity at point 75% of the flow-volume loop; MEF_75_, % pred—relative maximum expiratory flow at point 75% of the “flow-volume” loop; MEF_25_, L/s—maximum expiratory velocity at point 25% of the “flow-volume loop”; MEF_25_, % pred—relative maximum expiratory velocity at the point 25% of the “flow-volume” loop; TNAF, cm^3^/s—total nasal airflow, RTNAF, % pred—relative total nasal airflow*.

The severity of BA symptoms, as well as nasal symptoms, was comparable in boys and girls in the study; the values of the ACQ-5 test corresponded to achieved and/or partial BA control. The median values of FVC (% pred), FEV_1_ (% pred), and MEF_75_ (% pred) were not reduced, but the median values of the FEV_1_/FVC ratio (%) were lower than 85%, and those for MEF_25_ (% pred) were lower than 70% for both boys and girls. This can be regarded as evidence of a certain limitation of bronchial patency, including for small bronchi, in the sample of patients with BA.

The median TNAF values had no gender differences and were 610.4 [565.7; 655.1] Pa/cm^3^/s in the total sample, which is higher than 500 Pa/cm^3^/s, and therefore, corresponds to normal values of nasal respiratory function (47). At the same time, relative values—RTNAF for girls were slightly higher than for boys, *p* = 0.06.

### The Relationship Between Nasal and Bronchial Patency in Children With BA

The results of evaluating the relationship between rhinomanometry indicators, which reflect nasal patency, and spirometry indicators, which reflect bronchial patency, as well as anthropometric indicators, are shown in Table 2. There is a direct statistically significant relationship between absolute values of TNAF (Pa/cm^3^/s), and absolute values of spirometric indicators, including FVC (l), FEV_1_ (l), MEF_75_ (l/s), and MEF_25_ (l/s), which is obviously due to the dependence of nasal and bronchial respiratory flows on the anthropometric indicators of patients, particularly height. Thus, in the study of respiratory nasal function, it is necessary to consider not only the absolute parameters of AARM but also the value of these indicators in relation to their expected values in accordance with the physical development of patients. As a result, it is important to introduce relative indicators of nasal respiratory function (% pred) similar to the parameters of spirometry. To evaluate nasal respiratory function taking into account the age, gender, and height of patients, we used the previously proposed methods for evaluating RTNAF (14). Data on the relationship between RTNAF and relative values of spirometric indicators (%pred) are shown in Table 3.

**Table 2:** The relationship of absolute values of total nasal airflow to height and spirometry parameters in children with BA.

**Parameters**	**FVC, l**	**FEV1, l**	**MEF75, l/s**	**MEF25, L/s**	**Height, cm**
TNAF, cm^3^/s	0.36 (0.0006)	0.39 (0.0002)	0.32 (0.003)	0.39 (0.0002)	0.39 (0.0002)

*The data is presented as R (p), where R is the correlation coefficient and p is the level of statistical significance*.

*TNAF, cm^3^/s—total nasal airflow; FVC, l—forced vital capacity of the lungs; FEV1, l—forced expiratory volume in 1 s; MEF75, l/s—maximum expiratory velocity at point 75% of the flow-volume loop, MEF25, l/s—maximum expiratory velocity at point 25% of the flow-volume loop*.

**Table 3:** Relationship of relative total nasal airflow (RTNAF, %pred) with relative spirometry parameters in children with BA.

	**FVC (%pred)**	**FEV_**1**_ (%pred)**	**FEV_**1**_/FVC (%)**	**MEF_**75**_ (%pred)**	**MEF_**25**_ (%pred)**
Cohort, *n* = 88	0.01 (0.95)	0.11 (0.30)	0.16 (0.16)	0.05 (0.64)	0.22 (0.04)

*The data is presented as R (p), where R is the correlation coefficient, and p is the level of statistical significance*.

*FVC, % pred—relative forced vital capacity of the lungs; FEV1, % pred—relative forced expiration volume in 1 s; MEF75, % pred—relative maximum expiratory flow at point 75% of the “flow-volume” loop; MEF25, % pred—relative maximum expiratory velocity at the point 25% of the “flow-volume” loop*.

We did not establish any relationship between the RTNAF index and such spirometry indicators as FVC (% pred), FEV_1_ (% pred), and MEF_75_ (% pred). The relationship between RTNAF and FEV_1_/FVC (%) was somewhat more pronounced, however, it was not statistically significant—*p* = 0.16. At the same time, a statistically significant relationship between RTNAF and MEF_25_ (% pred) was identified. This suggests that the patency of small airways is most dependent on nasal respiratory function, and this should be taken into account when managing these patients (48).

Taking into account the observed tendency to the presence of gender differences in RTNAF described above in the clinical characteristics of children, the relationship of AARM parameters (Pa/cm^3^/s and % pred) to absolute and relative spirometric indicators in girls and boys were analyzed (Tables 4, 5).

**Table 4:** Correlation of absolute values of total nasal airflow to height and spirometry parameters in boys and girls with BA.

**Parameters**	**Height, cm**	**FVC, l**	**FEV_**1**_, l**	**MEF_**75**_, l/s**	**MEF_**25**_, l/s**
Boys, *n* = 58	0.53 (*p* < 0.0001)	0.47 (0.0002)	0.50 (0.0001)	0.42 (0.0009)	0.51 (*p* < 0.0001)
Girls, *n* = 30	0.19 (0.30)	0.23 (0.21)	0.21 (0.26)	0.09 (0.62)	0.12 (0.52)

*The data is presented as R (p), where R is the correlation coefficient and p is the level of statistical significance*.

*TNAF, cm^3^/s—total nasal airflow; FVC, l—forced vital capacity of the lungs; FEV_1_, l—forced expiratory volume in 1 s; MEF_75_, l/s—maximum expiratory velocity at point 75% of the “flow-volume” loop; MEF_25_, l/s—maximum expiratory velocity at point 25% of the “flow-volume” loop*.

**Table 5:** Relationship of relative indicators of total nasal airflow (RTNAF, %pred) to relative parameters of spirometry in children with BA, taking into account the gender of patients.

	**FVC (%pred)**	**FEV1 (%pred)**	**FEV1/FVC (%)**	**MEF 75 (%pred)**	**MEF25 (%pred)**
Boys, *n* = 58	−0.07 (0.60)	0.09 (0.52)	0.16 (0.23)	0.11 (0.40)	0.28 (0.03)
Girls, *n* = 30	0.07 (0.71)	0.08 (0.67)	0.07 (0.71)	−0.04 (0.85)	0.07 (0.71)

*The data is presented as R (p), where R is the correlation coefficient and p is the level of statistical significance*.

*RTNAF, % pred—relative total nasal airflow; FVC, % pred—relative forced vital capacity of the lungs; FEV_1_, % pred—relative forced expiratory volume in 1 s; MEF_75_, % pred—relative maximum expiratory flow at point 75% of the “flow-volume” loop; MEF_25_, % pred—relative maximum expiratory flow at point 25% of the “flow-volume” loop*.

It was found that boys had a direct statistically significant relationship between TNAF (Pa/cm^3^/s) levels and FVC (l), FEV_1_ (l), MEF_25_ (l/s), and MEF_25_ (l/s), which is probably due to the influence of the children's level of physical development on these indicators. This is confirmed by the presence of a statistically significant relationship between TNAF and child height. At the same time, girls in this sample did not show a statistically significant relationship between absolute TNAF indicators and spirometry parameters or anthropometric indicators (Table 4).

A comparison of the RTNAF index with spirometry indicators normalized in relation to expected spirometric values, allowed us to establish a direct correlation between RTNAF and MEF_25_ (% pred) only among the boys, which was more pronounced than in the general group (Table 5). With the girls, however, no relationship between RTNAF and any of the analyzed relative spirometry indicators, characterizing bronchial patency, was identified.

## Discussion

In this research, the functional relationships between the upper and lower respiratory tracts in children with BA and nasal symptoms were studied. With regard to this, we carried out an objective assessment of nasal obstruction using rhinomanometry, and an assessment of bronchial patency using such spirometric indicators as FEV_1_, the Tiffneau index, evaluation of flow at points 75 and 25% of the flow-volume curve of forced vital capacity.

It was found that in the sample of patients with BA and nasal symptoms, there is a statistically significant direct correlation between the absolute values of nasal inspiratory airflow and spirometry parameters that characterize bronchial patency. However, in our opinion, this cannot reflect the dependence of both spirometric and rhinomanometric indicators on the physical development of patients. The relationship between AARM indicators and anthropometric parameters was also demonstrated in this study. Previously, similar results were obtained in the works of Vig and Zajac (49), as well as Samolinski et al. (50).

To evaluate the personal characteristics of air flow in the lower respiratory tract, a comparison of spirometry indicators of patients with that of healthy individuals, with similar characteristics of age, gender, height, and race (% pred), is used. It is obvious that such an approach is justified to assess rhinomanometry indicators as well. However, while there are unified population data for spirometry indicators, for AARM indicators, there are only few publications summarizing the results of nasal respiratory function studies in a limited number of healthy individuals (30–32). This makes it difficult to compare the results of rhinomanometry of patients with indicators predicted based on the age, gender, and height of patients (% pred). To overcome this hurdle, we use the RTNAF index introduced and tested earlier (14), which takes into account the age, gender and anthropomorphic differences using the reference indicators (AARM data for healthy children) obtained by Zapletal et al. (31).

This allowed us to evaluate the association between nasal and bronchial patency in children with BA, using relative indicators (% pred) of both AARM and spirometry. Our study did not reveal a statistically significant relationship between TNAF and such classic spirometry indicators that characterize airflow limitation as: FEV_1_, FEV_1_/FVC index, MEF_75_ of the flow-volume loop, which reflects the flow rate in the proximal quartile of the forced vital capacity. At the same time, we established a statistically significant direct relationship between RTNAF and MEF_25_ (% pred) of the flow-volume loop, which characterizes the flow rate in the distal quartile of forced vital capacity, and demonstrates bronchial patency at the level of small bronchi. This suggests that nasal obstruction may be associated with airway limitation in small bronchi, the pathological process in which has been the focus of attention of researchers in BA, in recent years (51). The mechanism of this particular association is unclear. Perhaps the small airways are more susceptible to inflammatory and remodeling processes (52, 53). It is specifically small airway dysfunction that is present in the vast majority of children suffering from BA (54). Small airways may be the most vulnerable and sensitive to both the rhinobronchial reflex and cytokinemia, which are currently being considered as the main mechanisms of the pathological influence of AR/ARS on the course of BA (17). However, this assumption requires further study, which would include the identification of local and systemic biomarkers of inflammation and remodeling (6, 55–57). Nevertheless, it is worth noticing that our data are consistent with the results of studies by Iyer and Athavale (34), which showed that patients with AR are characterized by a high risk of latent damage to the small respiratory tract. The data we obtained partially agree with the results of Motomura and co-authors. These authors reported simultaneously lower indicators of nasal and bronchial patency in children with BA, who had a pale nasal mucosa, as compared to patients who had normal nasal mucosa color (33). Previously, Ciprandi et al. (26) found a direct correlation between FEV1 and nasal airflow in adult patients. In our study, no significant correlation was found between these parameters, which can be explained by the difference in spirometric characteristics of adults and children with asthma.

In addition, it should also be noted that in our study, certain gender differences were identified. They consisted in the fact that at comparable values of ACQ-5, relative spirometry indicators (%pred), and all *p* > 0.2, there was a tendency toward lower indicators of nasal respiratory function in boys compared to girls. In the available sample, RTNAF for boys was 89.9 [82.3; 97.5]%, and for girls−103.4 [90.0; 116.8]%, *p* = 0.06. Also in this study, among boys, as in the general cohort of patients, a direct statistically significant relationship was found between absolute values of TNAF (Pa/cm^3^/s) and the studied spirometry indicators, such as FVC (l), FEV1 (l), MEF_75_ (l/s), MEF_25_ (l/s), all *p* < 0.001. In boys, there was also a statistically significant direct relationship between RTNAF and MEF_25_ (% pred), *R* = 0.28, *p* = 0.03. At the same time, none of these relationships was established in girls.

On one hand, this may be due to the fact that there was a predominance of boys in the sample of patients under consideration. However, our results are consistent with data from other researchers, such as Samolinski et al. (50), who also note the presence of gender differences in nasal respiratory function.

Perhaps we are dealing with a modification of allergic inflammation under the influence of neuroendocrine activity. In a previous study, we demonstrated statistically significant differences in the composition of the nasal microbiota in boys and girls with BA and AR (58). This is important because the pathological airway microbiota changes can contribute into the pathogenesis of respiratory allergy (59). Many researchers note the presence of clinical and morphological differences in the pathology of the nose and paranasal sinuses in patients of different genders. Thus, a study by Bernstein et al. (60) found that sinonasal polyposis was more common among male patients than in women. In a study by Busaba et al. (61), it is noted that males complain more of nasal breathing difficulties, while females typically complain of headaches. It can be assumed that neuroendocrine influences may have different effects on the functional state of the respiratory tract, as evidenced by studies indicating gender differences in the prevalence of respiratory allergies, especially asthma in different age periods (61, 62).

The mechanisms underlying these differences include the effect of female hormones on the immune response and, in particular, the modulation of the inflammatory response by estrogens (63). Regarding sexual dimorphism, experimental studies have demonstrated gender differences in the expression profiles of mast cell histamine receptors and type 2 cytokine synthesis (64). In the study by Roved et al. (65), it was demonstrated that estrogen and progesterone increase type 2 and suppress type 1 reactions in women, while testosterone suppresses type 2 reactions, but has an uneven structure for type 1 reactions in men.

Undoubtedly, the nature of the relationship between nasal respiratory function and spirometric parameters in girls with BA requires further research, among other things, in comparison with the endocrine profile of patients.

## Conclusion

Significant positive correlation of absolute values of rhinomanometric and spirometric parameters in children with BA apparently, reflects the physical development of children. Among the relative spirometry indicators, only MEF_25_ (% pred), which reflects the patency of small bronchi, has a clear direct correlation with RTNAF. Hence, it can be assumed that the permeability of the small airways in children with asthma is greatly dependent on nasal respiratory function. These patterns are clearly expressed in boys with BA. In girls with asthma, the relationship between nasal respiratory function and spirometric indicators appears to be more complex and requires additional research, including comparison with the endocrine profile of patients.

## Data Availability Statement

The raw data supporting the conclusions of this article will be made available by the authors, without undue reservation.

## Ethics Statement

The study was approved by the Ethics Committee of the Privolzhsky Research Medical University, Protocol No. 13 of 10.10.2016. Informed consent was obtained from patients aged 15–17 years, and from the parents of patients under the age of 15 years, in accordance with Federal law No. 323 of 21.11.2011 On the basics of health protection of citizens in the Russian Federation. Written informed consent to participate in this study was provided by the participants' legal guardian/next of kin.

## Author Contributions

TE and DO conceived in the study and including design. SK, AN, and AS did the otolaryngological and functional examinations of patients. AK and RK were responsible for collecting and interpreting data, were performed. NK and MD-A conducted the statistical analysis. All authors have read and approved the final manuscript.

## Conflict of Interest

The authors declare that the research was conducted in the absence of any commercial or financial relationships that could be construed as a potential conflict of interest.
